# Recombinant Minimalist Spider Wrapping Silk Proteins Capable of Native-Like Fiber Formation

**DOI:** 10.1371/journal.pone.0050227

**Published:** 2012-11-28

**Authors:** Lingling Xu, Jan K. Rainey, Qing Meng, Xiang-Qin Liu

**Affiliations:** 1 Institute of Biological Sciences and Biotechnology, Donghua University, Shanghai, P.R. China; 2 Department of Biochemistry and Molecular Biology, Dalhousie University, Halifax, Nova Scotia, Canada; 3 Department of Chemistry, Dalhousie University, Halifax, Nova Scotia, Canada; University of Crete, Greece

## Abstract

Spider silks are desirable biomaterials characterized by high tensile strength, elasticity, and biocompatibility. Spiders produce different types of silks for different uses, although dragline silks have been the predominant focus of previous studies. Spider wrapping silk, made of the aciniform protein (AcSp1), has high toughness because of its combination of high elasticity and tensile strength. AcSp1 in *Argiope trifasciata* contains a 200-aa sequence motif that is repeated at least 14 times. Here, we produced in *E. coli* recombinant proteins consisting of only one to four of the 200-aa AcSp1 repeats, designated W_1_ to W_4_. We observed that purified W_2_, W_3_ and W_4_ proteins could be induced to form silk-like fibers by shear forces in a physiological buffer. The fibers formed by W_4_ were ∼3.4 µm in diameter and up to 10 cm long. They showed an average tensile strength of 115 MPa, elasticity of 37%, and toughness of 34 J cm^−3^. The smaller W_2_ protein formed fewer fibers and required a higher protein concentration to form fibers, whereas the smallest W_1_ protein did not form silk-like fibers, indicating that a minimum of two of the 200-aa repeats was required for fiber formation. Microscopic examinations revealed structural features indicating an assembly of the proteins into spherical structures, fibrils, and silk-like fibers. CD and Raman spectral analysis of protein secondary structures suggested a transition from predominantly α-helical in solution to increasingly β-sheet in fibers.

## Introduction

Spider silks are promising biomaterials with many potential uses in medicine, materials science and other fields, because of their exceptionally high tensile strength, elasticity, and toughness. For example, dragline silk can exhibit toughness surpassing even the strongest synthetic fibers, including nylon, Kevlar, and high-tensile steel [1]. Spider silks, because they are made of protein, are also biocompatible and biodegradable. These features make spider silks highly desirable for medical applications such as tissue engineering and drug delivery. For example, spider silk fibers have been described as being promising biomaterials for a biocompatible artificial nerve conduit [2] and for the enhancement of skin regeneration [3]. Before such potential uses can be fully realized, effective means of spider silk production and functional application are required, which may be achieved through better understanding of spider silk proteins and the mechanisms of silk fiber formation.

Production of spider silks from recombinant proteins is highly preferred to harvesting them from spiders. In particular, spiders are cannibalistic and consequently cannot be farmed like silkworms. Furthermore, the use of recombinant proteins permits desirable modifications to be introduced through genetic engineering and other means. However, full-length spider silk proteins are extremely difficult to produce in microorganisms, due to their large sizes and highly repetitive amino acid sequences. This difficulty has been attributed to factors including genetic instability, tRNA exhaustion, and mRNA secondary structure [4]. *Escherichia coli* (*E. coli*) is a preferred low-cost host cell for protein production, but has only been shown to be able to produce small fragments of spider silk proteins with relatively high yields. Some progress has been made in overcoming this difficulty through codon optimization and by employing different protein expression systems. Recently, a native-sized dragline silk protein (284.9 kDa) was expressed in a modified *E. coli* strain, although the expression level was relatively low [5]. Several eukaryotic systems, including yeast, plants, and cultured insect or mammalian cells, can also be used to express recombinant spider silk proteins [6], [7], [8], [9]; however, protein expression in these systems has typically been limited by low yields and/or high cost.

A better understanding of the mechanisms of silk fiber formation is also needed in order to produce useful silk-like fibers from recombinant spider silk proteins. Small fragments of spider silk proteins usually have failed to form silk-like fibers, although some of them have been observed to form microscopic fibrils [10], [11], [12], [13], [14]. A relatively large fragment (60–140 kDa) of a dragline silk protein, which was produced in cultured mammalian cells, was amenable to being spun into silk-like fibers [15]. More recently, a native-sized dragline silk protein (284.9 kDa) was produced in *E. coli* and spun into silk-like fibers with high strength [5]. However larger silk proteins are more difficult to produce at high yield and low cost, and the spinning process usually has employed organic solvents and has been less suitable for the studying of the molecular mechanisms of fiber formation. Interestingly, a relatively small fragment (4RepCT, 23.8 kDa) of the dragline silk protein of *Euprosthenops australis* was found to self-assemble in a physiological buffer to form silk-like fibers [16] which provided a unique system for studying the mechanisms of the silk formation. In particular, the C-terminal conserved non-repetitive domain of dragline silk protein was found to play several essential roles in silk formation, which included switching the silk protein to the assembly forms and aligning certain structural elements of the repetitive core domains of the silk protein [17], [18]. Two competing models for silk fiber formation have been advanced, with one requiring formation of a liquid-crystalline phase (the “liquid crystal” theory) and an the alternative involving the formation of proteinaceous micelles or spheres [19].

Spiders can produce six types of silk fibers, which differ greatly in physical properties and in protein content [20], [21], [22]. Previous studies have primarily focused on dragline silk because of its high tensile strength. Spider silks have been categorized into three major groups, based on the properties of their constituent protein sequences [23]. The most frequently studied group is represented by dragline silk, whose proteins (MaSp1 and MaSp2) are characterized by having small sequence motifs that are repeated a hundred times or more. These small motifs include polyalanines that are thought to form β-sheet crystallites responsible for the high tensile strength of the fiber, in addition to GGX and GPGXX motifs [1]. Another group is represented by flagelliform silk, which has the highest elasticity among spider silks. Its protein sequence contains small repetitive GGX, GPGGX, and GPGGAGGPY motifs but lacks the polyalanine motif [24]. Spider wrapping silk (aciniform silk) represents the third distinctive group. Its protein sequence contains repetitive domains that are much larger and highly homogeneous and lacks the small sequence motifs of the dragline and flagelliform silk proteins [25]. These large differences in primary structure are likely to have a strong influence upon the different physical properties of each of the spider silks, and they may lead to different structural and mechanistic features of silk formation. Consequently, it is important to study and compare the different groups of spider silk proteins.

Spider wrapping silk, also known as aciniform silk, has the highest toughness of the spider silks and is renowned for its ability to absorb energy without failing, because of a combination of high tensile strength and high elasticity (extensibility) [25]. Spiders use wrapping silk to wrap and immobilize prey and to build sperm webs and web decorations. The wrapping silk of *Argiope trifasciata* has been shown to be approximately 50% tougher than even dragline silk. Its protein, referred to as AcSp1, is produced in the aciniform gland and is predicted to be at least 280 kDa in size [25]. The AcSp1 protein sequence, which has been predicted from an incomplete gene sequence, contains a large core domain that is made up of repetitive sequences, and a small C-terminal domain that is non-repetitive and conserved among different spider silk proteins. It is not known whether AcSp1 contains a conserved N-terminal non-repetitive domain found in other spider silk proteins, because this portion of the AcSp1 gene sequence was not determined. The large core domain contains a 200-aa repeat that is repeated at least 14 times and is highly homogeneous. The 200-aa repeat of AcSp1 is very different from the repeats of other spider silk proteins in size and sequence. For example, the 200-aa repeat has a lower content (∼25%) of Gly and Ala, compared to those of dragline silk proteins and flagelliform silk protein, which typically have a Gly and Ala content of ∼ 55% and ∼52%, respectively [25]. These unique features of the AcSp1 protein, together with the exceptional physical properties of spider wrapping silk, make the AcSp1 protein an interesting subject for studying structure-function relationships of recombinant spidroin as well as the mechanisms of silk formation.

In this study, we have for the first time produced and studied recombinant wrapping silk proteins. Recombinant proteins consisting of only the 200-aa repeats of AcSp1 were produced in *E. coli* with relatively high yields and fused with a removable protein tag for affinity purification. We found that as few as two of the 200-aa repeats were sufficient to form silk-like fibers when induced by shear forces in a physiological buffer, without needing a conserved C-terminal non-repetitive domain. These fibers exhibited small diameter and high extensibility. Examinations using microscopic and biophysical techniques revealed protein structural changes and possible intermediates of fiber formation.

## Materials and Methods

### Construction of Recombinant Plasmids

To construct the plasmid vector pEHU, a PCR-amplified DNA coding for a hexahistidine tag (H_6_-tag) fused to a SUMO protein was inserted into the pET32 plasmid (New England Biolabs, Ipswich, USA) between restriction sites *Nde*I and *Bam*HI. Two additional restriction sites (*Bsa*I and *Bfu*AI) were introduced after the SUMO coding sequence through inverse PCR, using two oligonucleotide primers: 5′-GAGGCGGTTAGCAGGTCAACACAGCTTATAC-3′ and 5′-GAGGTCTCTCCAGCTCCACCAATCTGTTCTCTGTG-3′, with the restriction sites being underlined.

**Figure 1:**
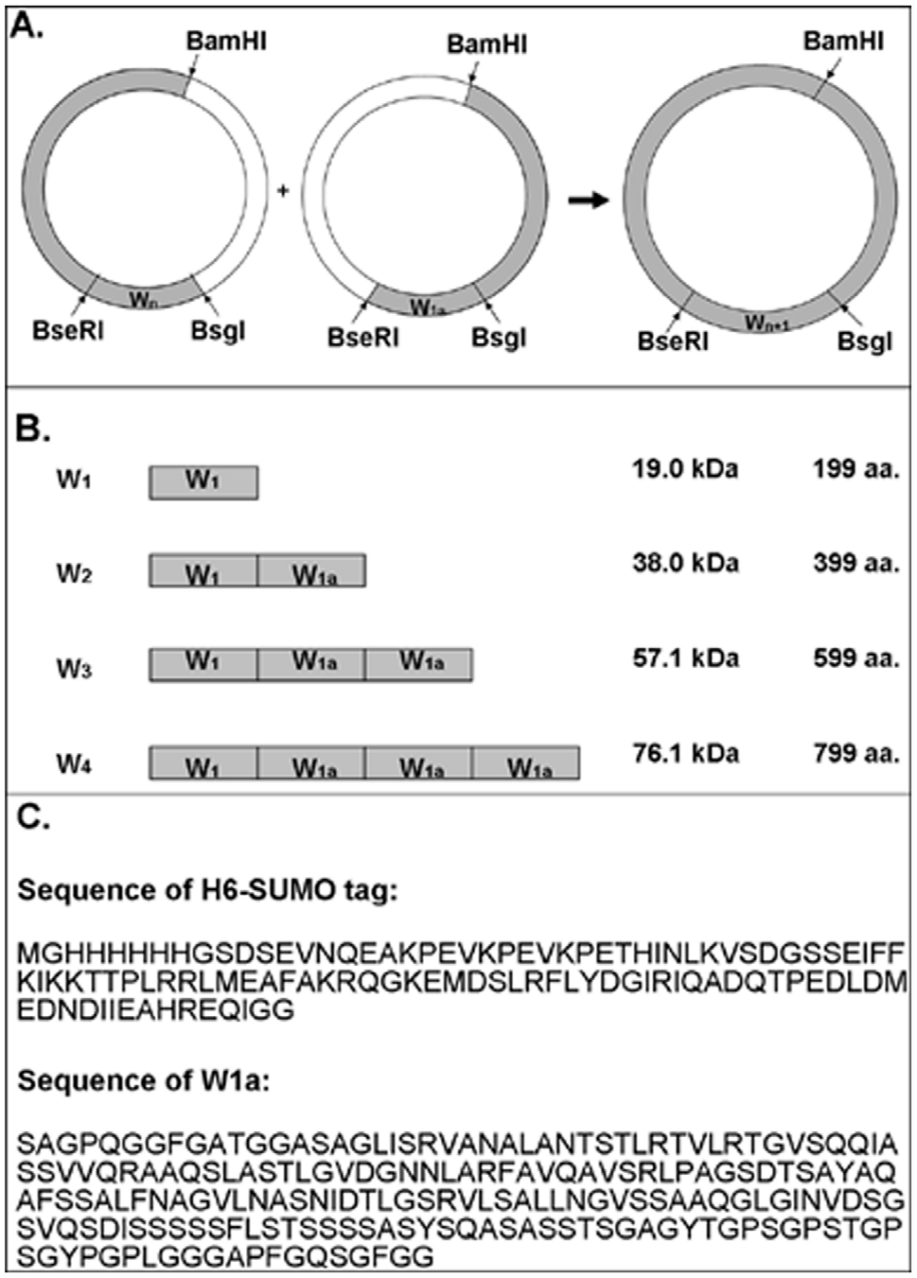
Construction of AcSp1-derived recombinant proteins. (A) Schematic illustration of construction strategy of the recombinant genes. Plasmid pW_n_ (n = 1, 2, or 3) was digested with restriction enzymes *Bam*HI and *Bsg*I, and the resulting *Bam*HI-*Bsg*I fragment containing the W_n_ coding sequence was ligated with a *Bam*HI-*Bse*RI fragment isolated from plasmid pW_1a_ containing the W_1a_ coding sequence. The resulting plasmid pW_n+1_ has the W_n_ and W_1a_ coding sequences seamlessly fused to produce the W_n+1_ coding sequence. (B) Schematic illustration of the W_1_ to W_4_ proteins with their sizes shown. (C) Amino acid sequences. The 106-aa H_6_-SUMO tag was added to the N-terminus of the W_1_ to W_4_ proteins. The 200-aa W_1a_ sequence was derived from the consensus repeat of the AcSp1 protein of *Argiope trifasciata* [25]. The 199-aa W_1_ sequence (not shown) was the same as W_1a_ except the absence of the N-terminal S residue.

Recombinant genes (W_1_ through W_4_) were constructed as follows. A coding sequence of the 200-aa repeat of AcSp1 was made as a synthetic gene (Integrated DNA Technologies, Coralville, Iowa). The 200-aa sequence was based on the consensus repeat sequence of *Argiope trifasciata* AcSp1, and its coding sequence was designed for optimal codon usage of *E. coli* without altering amino acid sequence. This coding sequence was inserted in the pDrive plasmid (New England Biolabs, Ipswich, USA) between restriction sites *Mlu*I and *Xba*I to produce plasmids pDW_1_ and pDW_1a_. In these plasmids the AcSp1 coding sequence was flanked with a 5′ sequence containing *Bse*RI and *Bsa*I sites and a 3′ sequence containing *Bfu*AI and *Bsg*I sites, pDW_1a_ differed from pDW_1_ by having 12 more nucleotides at the 5′ end, which were designed for seamless fusion of multiple repeat coding sequences. To construct genes coding for two or more of the 200-aa repeats, a previously reported cloning strategy [24], [26] was used as follows (illustrated in Figure 1A). Plasmids pDW_1_ and pDW_1a_ were digested separately to produce a *Bam*HI – *Bsg*I fragment and a *Bam*HI – *Bse*RI fragment, respectively, and ligation of these two DNA fragments produced plasmid pDW_2_ coding for two repeats. To make plasmid pDW_3_, which coded for three repeats, pDW_2_ and pDW_1a_ were digested to produce a *Bam*HI – *Bsg*I fragment and a *Bam*HI – *Bse*RI fragment, respectively, and ligation of these two DNA fragments produced pDW_3_. To make plasmid pDW_4_, which coded for four repeats, pDW_3_ and pDW_1a_ were digested to produce a *Bam*HI – *Bsg*I fragment and a *Bam*HI – *Bse*RI fragment, respectively, and ligation of these two DNA fragments produced pDW_4_.

To construct the expression plasmid pEHU-W_1_, a 594 bp *Bsa*I - *Bfu*AI DNA fragment was isolated from plasmid pDW_1_ (see above) and inserted in plasmid pEHU (see above) between the same two sites. Similarly, expression plasmids pEHU-W_2_ pEHU-W_3_ and pEHU-W_4_ were constructed by isolating *Bsa*I - *Bfu*AI fragments from plasmids pDW_2_, pDW_3_, and pDW_4_, respectively, and inserting the fragments into plasmid pEHU between the same two sites. Note that the cutting sites of restriction enzymes *Bsa*I, *Bsg*I, *Bse*RI, and *Bfu*AI are outside of their recognition sequences and this permitted seamless fusion of the coding sequences.

### Protein Expression and Purification

Each expression plasmid was transformed into *E. coli* BL21 (DE3) (Novagen, Darmstadt, Germany) using standard protocols. Cells were grown at 37°C in Luria-Bertani medium containing ampicillin (50 µg/ml) to mid-log phase (OD_600_ of 0.8∼1.2) and then induced at room temperature overnight with 0.8 mM IPTG (isopropyl *β*-D-thiogalactoside) to stimulate expression of the plasmid-encoded fusion protein. Cells were harvested by centrifugation and resuspended in a lysis buffer (10 mM Tris-HCl, 300 mM NaCl, 20 mM imidazole, pH 8.0) and lysed using a French Pressure Cell Press (American Instrument Company). The cell lysate was centrifuged at 12,000 rpm for 15 minutes at 4°C to remove any insoluble cell debris, and the resulting supernatant was loaded onto a non-denaturing column packed with Ni-NTA Sepharose (Qiagen, Germany). The column was washed according to manufacturer’s instructions before the bound proteins were eluted in an elution buffer (10 mM Tris-HCl, 300 mM NaCl, 250 mM imidazole, pH 8.0).

The purified fusion protein was treated with SUMO protease to remove the H_6_-SUMO tag. Specifically, the fusion protein was mixed with the protease at a 100∶1 ratio (w/w) and dialyzed against a reaction buffer (10 mM Tris-Cl, pH 8.0) at 4°C overnight, which not only allowed the protease to cleave the fusion protein site-specifically after the SUMO sequence but also removed imidazole from the reaction mixture. Subsequently, the reaction mixture was passed through a column of Ni-NTA Sepharose, the tag-free recombinant protein flowed through the column and was collected as purified protein, while all other proteins (H_6_-SUMO tag, SUMO protease, and any remaining fusion protein) containing a H_6_-tag remained bound to the column. Protein samples were analyzed by SDS-PAGE and visualized by staining with Coomassie Brilliant Blue R-250. Protein concentration was obtained by comparing the intensity of protein bands to a standard protein marker (Fermentas) after drying and scanning SDS-PAGE gels, using Image J software.

### Identification of Purified W_1–4_ Proteins by Mass Spectroscopy

Samples were desalted by dialysis and then analyzed by Electrospray ionization mass spectrometry (ESI-MS). All mass spectra were acquired on a Waters Q-Tof Premier (Milford, MA) equipped with a nano-electrospray source. Samples were diluted to approximately approximately 20 ng/µl for W_1_, W_3_ and W_4_, and 40 ng/µl for W_2_ in 50% acetonitrile with 0.1% formic acid and infused at 5 µl/min. The mass spectrometer was scanned from 100 to 4000 m/z with a scan time of 1 second in TOF MS mode. A potential of 3000V was applied to a 30 µm id tapered electrospray tip. Cone voltage was set to 30 kV and source temperature set to 100°C. Data acquisition was performed using Masslynx version 4.1 (Waters Milford, MA).

### Fiber Pulling Procedures

Silk-like fibers were pulled from solutions of purified and tag-free W_2_ to W_4_ proteins in a buffer (10 mM Tris.HCl, pH 8.0) at room temperature. Typically, a 20-µl protein solution (∼0.4 mg/mL) was placed on a glass slide at room temperature, and fibers were pulled from the protein solution using a plastic 200 µl pipette tip, where one end of the fiber apparently attached to the pipette tip. Each pulling action was a continuous motion at a speed of ∼6 mm.s^−1^.

### Mechanical Tests of Silk Fibers

The diameter of each fiber was measured using light microscopy before testing its tensile strength. For each fiber, three digital photographs were taken at 1000× magnifications, with one each taken near the two ends and also the middle of the fiber. From each photograph, three locations were analyzed using Image Tool 2.0 software to determine the diameter of the fiber. The resulting nine diameter estimates for each fiber were averaged. The cross-sectional area of each fiber was calculated, assuming that the fibers were circular in cross-section. After excluding fibers that either showed uneven thickness or knots under the light microscope or produced an incomplete stress-strain curve, average mechanical properties of W_4_ was acquired from ten fibers.

Tensile strengths of the fibers were measured at 22.0±2°C and ∼40% humidity, using an Agilent T150 UTM with a nanomechanical actuating transducer (Agilent technologies, USA). Fibers were extended at a constant rate of 1% strain/s, relative to their original length, until they broke. Force and extension data were used to calculate the engineering stress and strain, respectively. Testworks 4.0 software (MTS Corp.) was used to visualize the stress-strain curves, to calculate stiffness (Young’s modulus E), and to calculate toughness by integrating the area under the stress-strain curve.

### Biophysical Characterizations of Proteins and Fibers

Circular dichroism (CD) spectra of protein solutions were recorded at 22±2°C, from 260 to 190 nm in a quartz cuvette (0.1 cm path length) using a J-810 spectropolarimeter (Jasco, Tokyo, Japan), with a scan speed of 20 nm/min, response time of 1 s, acquisition interval of 1 nm, and bandwidth of 2 nm. The samples were prepared in 50 mM phosphate buffer, pH 7.5, with protein concentrations of ∼0.06 mg/mL. A blank solution was measured under the same experimental conditions and subtracted from the data. Three scans were acquired and averaged for each sample.

Scanning electron microscopy (SEM) was performed using a Hitachi Cold Field Emission S-4700 Scanning Electron Microscope. For imaging protein solution structure, a 5-µl protein sample (in 10 mM Tris-Cl, pH 8.0) was placed on a cover slip and allowed to sit for 15–30 minutes, in order for the proteins to settle onto the surface which had been coated with poly-L-lysine. Subsequently the sample was initially fixed with a 2.5% glutaraldehyde solution (in 0.1M sodium cacodylate buffer) for 2 hours, rinsed three times (10 minutes each) with a 0.1M Sodium cacodylate buffer, and secondarily fixed with 1% osmium tetroxide for 2 hours, rinsed with distilled water, and dehydrated in a graded series of ethanol. After the ethanol was removed during the critical point drying, the sample was coated with gold particles by SC7620 mini sputter coater before SEM analysis. For imaging fiber cross section, W_3_ fibers were glued on paper frames which have gaps of ∼1 mm. Fibers were broke in liquid nitrogen by folding the paper frames and causing them to break at the gaps. Fibers with breaking ends were fixed on SEM stub by gluing the paper frames on the stub with an angle of ∼ 45° and were coated by gold particles before SEM analysis.

Raman spectra of silk fibers were recorded with an inVia Raman microscope (Renishaw) at 21.0±2°C and 30±5% RH. The 632.8 nm line of a He-Ne laser was used, and the laser beam was focused down with a 100× objective to a diameter of approximately 2 µm, generating an intensity of 6 mW at the sample. The spectra were corrected for a slight fluorescence background over the spectral range of 400–1800 cm^− 1^ using a polynomial baseline. For each sample, two independent fibers and multiple positions on each fiber were tested to assess sample uniformity.

## Results

### Fibers Formed from Recombinant Proteins Derived from AcSp1

The aciniform silk protein AcSp1 of *Argiope trifasciata* contains a 200-aa repeat that is highly homogeneous and reiterated at least 14 times [25]. We produced recombinant proteins consisting of one to four of the 200-aa consensus repeat of AcSp1 and designated them W_1_ to W_4_ (W = wrapping silk), respectively (Figure 1). A H_6_-SUMO tag was added to the N-terminus of each protein to allow affinity purification and subsequent tag removal was performed. To express these fusion proteins in *E. coli*, recombinant plasmids were constructed as illustrated in Figure 1. In this process, a previously reported cloning strategy [26] was used to assemble the repetitive coding sequences in a seamless way, and the coding sequence was designed for optimal codon usage in *E. coli* without altering amino acid sequence. Each plasmid was transformed into *E. coli* cells to express the fusion protein in a 200 ml cell culture.

**Figure 2:**
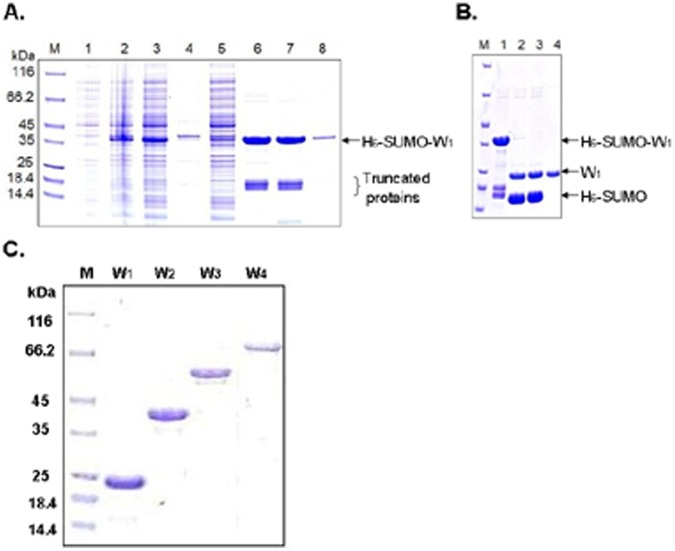
Protein expression and purification. Each fusion protein was expressed in *E. coli* and analyzed by SDS-PAGE followed by Coomassie blue staining, with protein size markers shown in lane M. (A) Expression and purification of the fusion protein H_6_-SUMO-W_1_. Lanes 1 and 2 are total cellular proteins before and after IPTG-induced protein expression, respectively. The induced cell lysate was separated into soluble (lane 3) and insoluble (lane 4) fractions, and the soluble fraction was passed through a nickel-beads affinity column. Lane 5 shows unbound proteins that flew-through the column, and lanes 6–8 are three consecutive fractions eluted from the column. (B) Removal of the H_6_-SUMO tag. The fusion protein H_6_-SUMO-W_1_ (lanes 1) was digested with a SUMO protease at 4°C for 6 hours (lane 2) and overnight (lane 3). The resulting sample was passed through a nickel-beads affinity column, and the tag-free W_1_ protein was collected as a purified protein in the flew-through fraction (lane 4). (C) The W_1_, W_2_, W_3_ and W_4_ proteins that were expressed and purified as in panes A and B.

**Figure 3:**
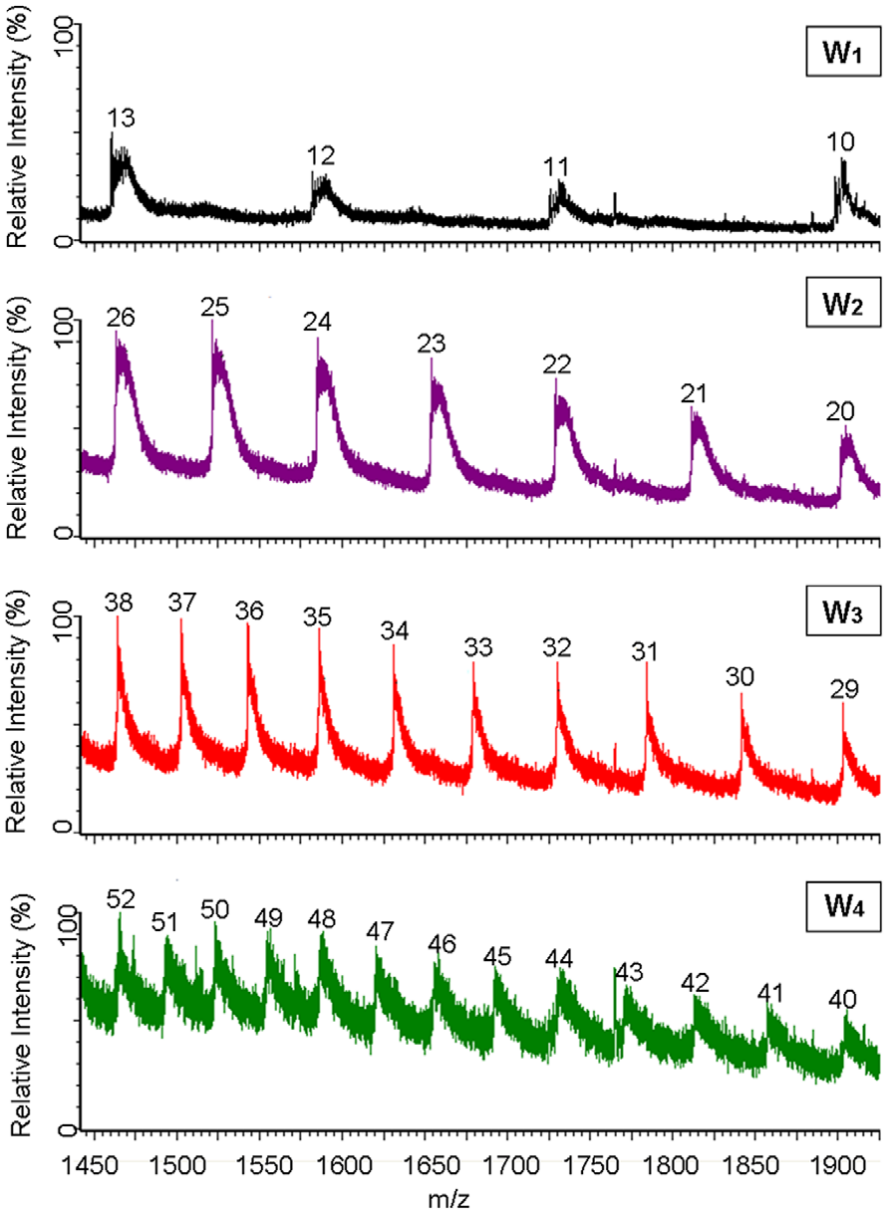
Electrospray ionization mass spectrum of W_1–4_. Numbers above each peak in blue are different charges states.

The protein expression levels ranged from ∼80 mg (for H_6_-SUMO-W_1_) to ∼22 mg (for H_6_-SUMO-W_4_) per liter of cell culture (Figure 2). All of the H_6_-SUMO-W_1_ and H_6_-SUMO-W_2_ proteins were soluble after cell lyses, whereas 60–80% of the H_6_-SUMO-W_3_ and H_6_-SUMO-W_4_ proteins remained soluble. Each fusion protein was purified from the soluble fraction of a cell lysate by affinity binding of the H_6_ tag to nickel beads and under non-denaturing conditions. This process routinely yielded approximately 10–40 mg of fusion protein from each liter of the *E. coli* cell culture, depending on protein size. The purified fusion proteins were stable at 4°C for at least one week without visible precipitation. When needed, the fusion protein was treated with SUMO protease at 4°C overnight to cleave off the H_6_-SUMO tag at the C-terminus of SUMO. When the cleavage products were subsequently passed through a Ni-column at 4°C, only the tag-free W_1_ to W_4_ proteins could flow through the column and be collected as purified proteins (Figure 2), while all of the other proteins (H_6_-SUMO tag, SUMO protease, any remaining fusion protein) contained a H_6_-tag and were therefore trapped on the column. The W_1_ to W_4_ proteins were purified to be 95% or better, based on intensities of the stained protein bands. The contaminating protein species could not be identified. The purified W_1_ to W_4_ proteins were confirmed to have the correct identity and full length through mass spectrometry analysis (Figure 3 and Table 1).

**Table 1:** Theoretical and observed average mass of W_1–4_.

Protein	Average Mass_theor_ (Dalton)	Average Mass_obs_ (Dalton)
W_1_	18975	18976
W_2_	38018	38011
W_3_	57062	57059
W_4_	76106	76131

Average Mass_theor_: theoretical average mass; Average Mass_obs_: observed average mass. Observed average mass was calculated from charge 7, 8 and 9 for W_1_, charge 16, 17 and 18 for W_2_; charge 30, 32 and 34 for W_3_; charge 44, 45 and 46 for W_4_.

**Figure 4:**
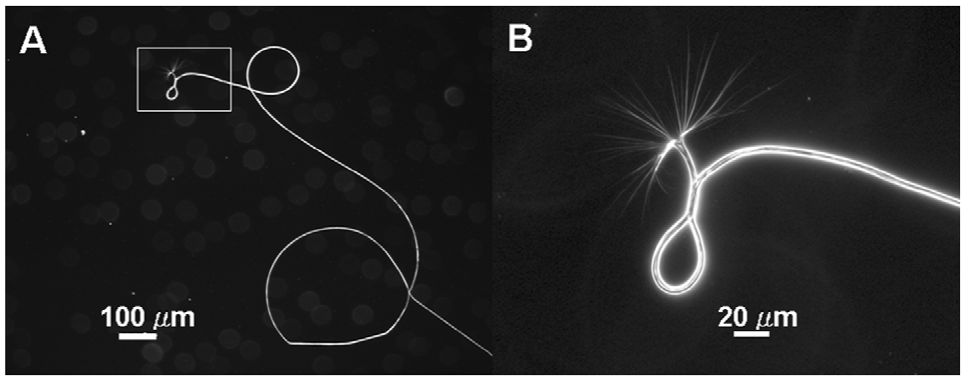
Appearances of the silk-like fiber. A fiber formed from the W_3_ protein was photographed under a light microscope. Panel B corresponds to the boxed area of panel A with a higher magnification.

Silk-like fibers pulled from solutions of purified W_2_ to W_4_ proteins ranged from approximately 1 to 10 cm long. They showed a homogeneously smooth surface and an average diameter of ∼3.4 micrometers, with a representative fiber shown in Figure 4. We tested different protein concentrations ranging from 0.04 to 0.6 mg/mL. The W_3_ and W_4_ proteins readily formed silk-like fibers at all concentrations. The W_2_ protein formed only shorter fibers (<2 cm) at lower protein concentrations (<0.1 mg/mL), and it formed much fewer fibers even at higher protein concentrations (>0.1 mg/mL) when compared to W_3_ and W_4_ proteins. We tested different incubation times, at room temperature, for the protein solutions on glass slides, before initiating the process of pulling silk-like fibers from the protein solution. Silk-like fibers could be pulled from the protein solution immediately, although more silk-like fibers could be pulled after a longer incubation time. We also tested lower temperatures and found that fibers could be formed at temperatures as low as 4°C. Silk-like fibers formed from the three proteins (W_2_, W_3_, W_4_) had similar diameters, and the diameters did not change significantly with different protein concentrations and incubation time. However, the W_1_ protein did not form silk-like fibers at any of the tested protein concentrations and temperatures, even after a prolonged incubation time, although this protein could form microscopic aggregates (spheroids) and nano-fibrils that are described later.

### Mechanical and Physical Properties of the Silk Fibers

**Figure 5:**
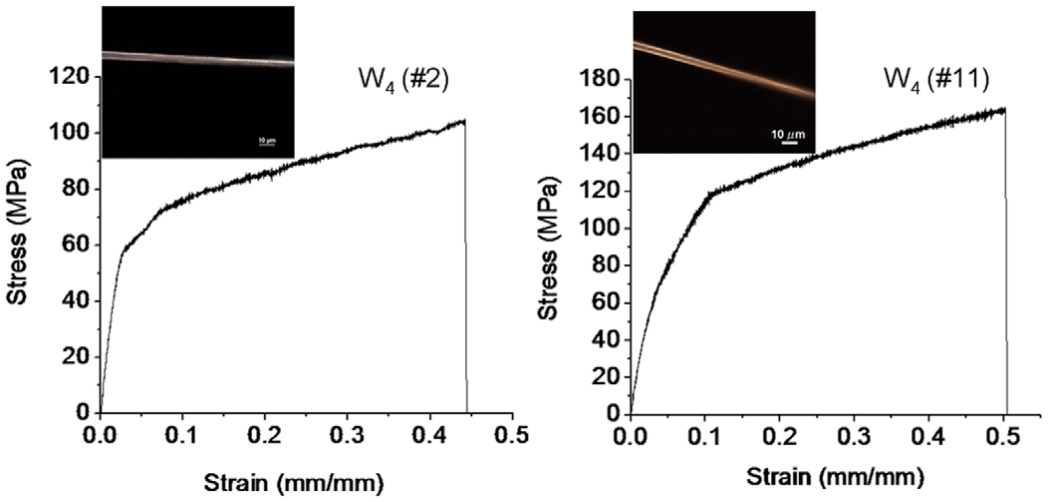
Stress-strain curves and light-microscopic images of two representative fibers formed from the W_4_ protein. The stress values are normalized to the initial cross-sectional area of the fiber. The strain corresponds to dL/L_0_, where L_0_ is the initial length of the fiber and dL is the change in fiber length.

Many of the silk fibers from W_4_ protein solution were sufficiently long (>3 cm) and uniform (without knots) to allow nanomechanical testing to be performed using standard instrumentation (e.g. Figure 5). The average values were obtained from ten fibers and compared directly to those of native and recombinant spider silk fibers in Table 2. The tensile strength of the W_4_ fibers was calculated to be ∼115 MPa, which is approximately one-sixth of the reported tensile strength of the natural wrapping silk of *Argiope trifasciata*. The average extensibility of the W_4_ fibers was calculated to be ∼37%, which is approximately half the reported extensibility of the natural wrapping silk. The average toughness of the W_4_ fibers was calculated to be ∼34 J cm^−3^. This is less than one-tenth of the reported toughness of the natural wrapping silk. The average diameter of the W_4_ fibers was ∼3.4 µm. This is approximately ten times larger than the diameter of natural wrapping silk.

**Table 2:** Comparison of physical properties of W_4_ fiber with those of other artificial and natural spider silk fibers.

Properties	Recombinant AcSp1 (W_4_)	Recombinant MaSp1 (4RepCT)	Recombinant MaSp1(native- sized)	Natural aciniform silk
Breaking strength (MPa)	115.06±24.44	80±20	508±108	687±56
Extensibility	0.37±0.11	0.01±0.001	0.15±0.05	0.86±0.03
Toughness (J cm^−3^)	33.83±13.45	–	–	376±39
Diameter (µm)	3.41±0.34	40–90	∼ 50	0.35±0.01

Properties of the recombinant AcSp1 (aciniform) fiber, which was formed from protein W_4_, were determined in this study. Properties of the recombinant MaSp1 (dragline) fiber, which was formed from protein 4RepCT, were obtained from [29]. Properties of the fiber formed from native sized MaSp1 (dragline) protein were obtained from [5]. Properties of natural aciniform silk were obtained from [25].

**Figure 6:**
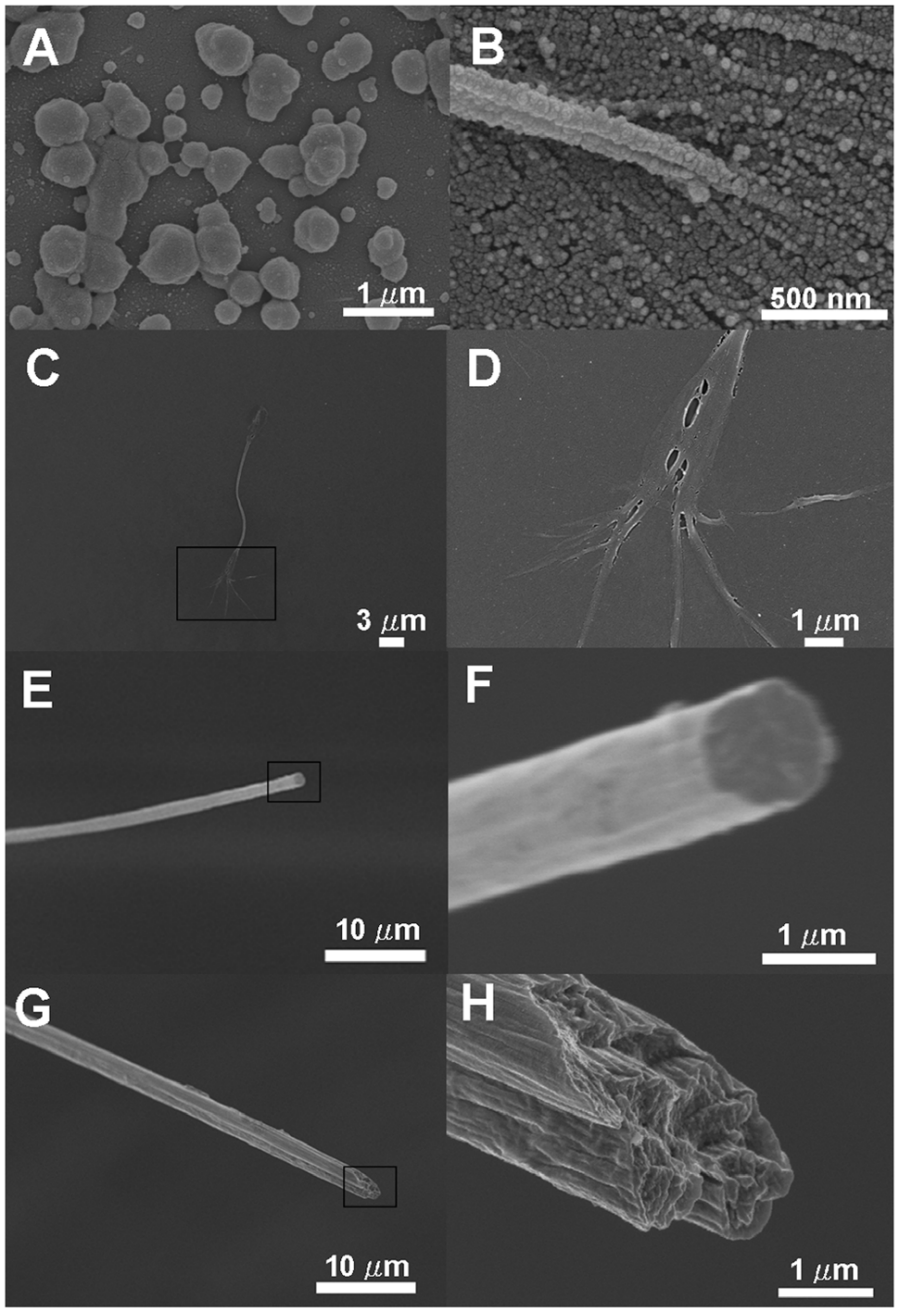
Scanning electron micrographs (SEM) of aggregates (spheroids) and fibrils/fiber formed from the W3 protein. The protein sample was immobilized either using a wet fixation method to preserve spheroids (panel A and B) or using a dry fixation method to retain fibrils (panel C and D). For panel B, the protein solution was swirled with a pipette tip to increase fiber formation before fixation. For imaging the macro-fiber, dry fibers were not treated by any solvent, but they were broken in liquid nitrogen to show fiber cross section (panel E to H). Panel D, F and H corresponds to the boxed areas of panel C, E and G, respectively, with a higher magnification.

To determine whether the W_3_ fibers have a solid or porous structure, the fibers were frozen and broken in liquid nitrogen, and the broken ends were examined with scanning electron microscopy (SEM). SEM images of two representative fibers are shown in Figure 6E–H, with one fiber having a flat broken end (Figure 6F) and another fiber having a slanted broken end (Figure 6H). These cross sections showed that the fibers are solid, not hollow or porous.

### Observation of Microscopic Fibrils and Spherical Aggregates in Fiber Formation

To find possible intermediate structures of the fiber formation, we examined fiber formation under a light microscope. A fiber was pulled, but not to completion, from a 5-μl solution of W_3_ protein on a microscope glass slide, with the tail end of the fiber still remaining in solution. By inverted light microscopy, the mature part of the fiber showed a smooth surface and a diameter of ∼4 μm (Figure 4A). The tail end of the fiber showed numerous smaller fibrils (Figure 4B).

To visualize smaller structures, the four protein samples (W_1_ to W_4_) were examined by scanning electron microscopy. Each protein solution was immobilized on a cover slip using two different fixation methods. The “wet fixation” method was employed to better preserve water-containing soft structures in its native state, but with the side effect that fiber materials would be lost during the washing steps. The “dry fixation” method was better at retaining fibers, but with the risk of destroying water-containing soft structures in the drying step. Some protein solutions were stirred (dragged on the sample slide) with a pipette tip several times in one direction to introduce shear forces with the goal of inducing fiber formation. For the W_2_, W_3_ and W_4_ proteins, an abundance of spherical structures and fibrils were observed, with some examples shown in Figure 6A–B for the W_3_ protein. The spheroids are not always round and have various sizes. Some spheres appeared to line up, with possible merging to form small fibrils (Figure 6B). Smaller fibrils were often observed at the ends of larger fibrils or fiber (Figure 6C, D). For the W_1_ protein, spheres were also observed abundantly and very small fibrils were seen occasionally, although the protein could not form silk-like fibers.

### Protein Structural Changes in Fiber Formation

**Figure 7:**
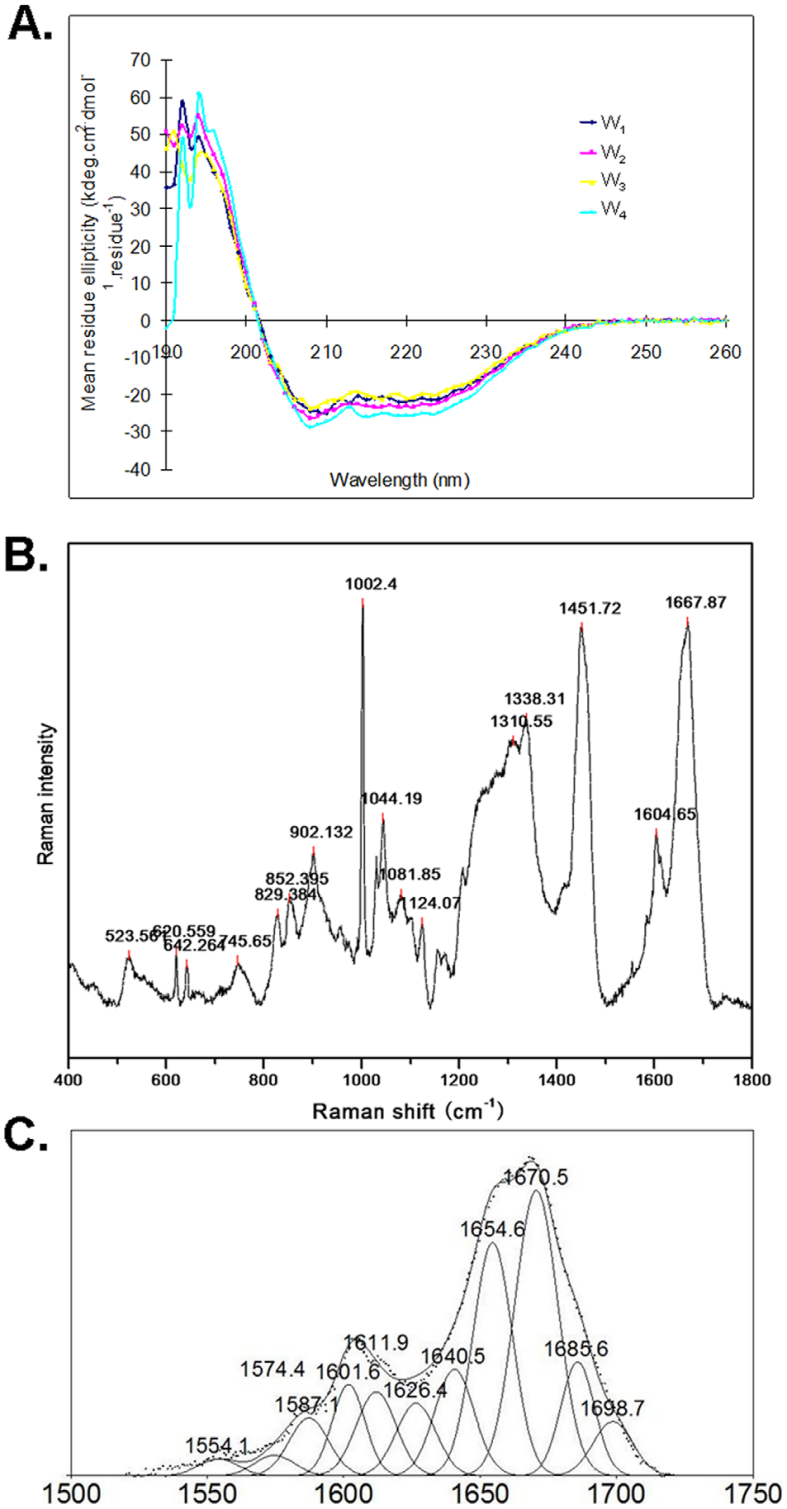
Analysis of protein secondary structures. (A) CD spectra of the W_1_, W_2_, W_3_, and W_4_ proteins in solution. (B) Raman spectra of a fiber formed from the W_4_ protein. (C) Spectral decomposition in the amide I region of the W_4_ silk.

During fiber formation, the recombinant proteins might have undergone structural changes to form β-sheets structure, because such structural changes had been known for some other silk proteins. To investigate this possibility, we first subjected the four recombinant proteins (W_1_ through W_4_) to circular dichroism (CD) analysis to reveal their secondary structure contents. The four proteins showed nearly identical CD spectra (Figure 7A), despite having large differences in size and fiber-forming ability. Each CD spectrum showed two negative bands at 208 and 220 nm, along with a positive band at 192 nm. This combination of bands is a known signature of α-helical structure. Therefore, the secondary structures of these proteins were predominantly α-helical when in solution before fiber formation. We then used Raman spectroscopy to reveal the secondary structure content of the silk-like fibers formed from W_4_ protein. The five fibers we analyzed gave nearly identical Raman spectra, with a representative spectrum shown in Figure 7B. After decomposition of the amide I band of the spectrum, α-helix and β-sheet are seen as the smaller peak at 1655 cm^−1^ and the larger peak at 1670 cm^−1^, respectively (Figure 7C), indicating that both α-helix and β-sheet structures are present in the fiber [23], [27].

## Discussion

We have produced a recombinant fragment of the spider AcSp1 protein capable of forming silk-like fibers. To our knowledge, this is the first study of a recombinant wrapping silk protein, with previous studies mostly focusing on dragline silk proteins. We determined the minimal size of the recombinant AcSp1 protein required for formation of the silk-like fibers. Recombinant proteins (W_2_ to W_4_) consisting of as few as two of the 200-aa repeats of AcSp1 were able to form silk-like fibers. This is significantly shorter in comparison to the native AcSp1 protein, which contains at least 14 of these repeats. A protein (W_1_) consisting of only one of the 200-aa repeats formed spherical structures abundantly and small fibrils occasionally, indicating that this small protein was also able to self-assemble, although the resulting microscopic structures did not form macroscopic silk-like fibers. It was somewhat surprising that the W_2_ to W_4_ proteins formed silk-like fibers without needing the C-terminal non-repetitive domain of the AcSp1 protein, considering that the C-terminal domain is conserved among different spider silk proteins and thought to play an important role in fiber formation under natural and some artificial conditions [17], [18], [28].

Fibers formed from recombinant protein W_4_ consisting of four of the 200-aa repeats of AcSp1 displayed silk-like properties, included fiber size, appearance, and toughness. The W_4_ fibers were up to 10 centimeters long, and this length was probably limited by the protein size and amount, because we noticed that longer fibers were more easily obtained from larger proteins (e.g. comparing W_4_ and W_2_ proteins), larger solution volumes, and higher protein concentrations. The W_4_ fibers showed a relatively uniform thickness (average diameter of ∼3.4 µm) when observed under microscope. The diameter of these artificial fibers is larger than the reported diameter (∼0.35 µm) of natural spider wrapping silk [25], probably due to the artificial conditions used in this study to form the fibers. The W_4_ fiber of this study showed a high tensile strength and elasticity, further suggesting a silk-like fiber. The average elasticity (extensibility, averaging 37%) of W_4_ fiber was nearly half of the reported extensibility (86%) of natural wrapping silk, and the average tensile strength (breaking strength, ∼115 MPa) reached one-sixth of the reported tensile strength (687 MPa) of natural wrapping silk. In comparison to artificial silk-like fibers formed from dragline silk recombinant proteins under different conditions [29], the W_4_ fiber had a much higher elasticity, although its tensile strength was lower than that of silk-like fibers spun from a native-sized dragline silk protein. Overall, physical properties of the W_4_ fiber were indicative of a silk-like fiber.

Formation of silk-like fibers from the AcSp1-derived recombinant proteins (W_2_, W_3_ and W_4_) indicated a shear-induced assembly of these proteins and may shed some light on the mechanism of fiber formation. The proteins clearly formed microscopic structures that may or may not reflect intermediate steps toward the formation of silk-like fibers. We observed small spherical structures and small fibrils of various sizes when the protein solution was analyzed under the electron microscope and the light microscope. Under some conditions, small spheres appeared to line up at the trailing end of fibrils, and small fibrils appeared at the trailing end of silk-like fibers. Previously spherical structures and small fibrils have also been observed with other recombinant spider silk proteins [10], [12], [30], however it is rare or unprecedented to observe spheres lining up behind fibrils and fibrils trailing behind silk-like fibers. Our findings suggest, but do not conclusively prove, that the spheres line up to form fibrils, and the fibrils might amalgamate to form silk-like fibers. Our findings therefore appear consistent with the previously proposed “micelle theory” for formation of silkworm silks [31], with the caveat that it is not known whether the spherical structures observed in our study are, strictly speaking, micelles.

The process of fiber formation from the AcSp1-derived recombinant proteins also involved changes in the secondary structures of the protein. Before forming fibers, the secondary structures of the proteins in solution appeared to be mostly α-helical, as indicated by CD spectroscopy. In the silk-like fibers, the protein’s secondary structure appeared to be a mixture of both α-helix and β-sheet, as indicated by the Raman spectra of the silk-like fibers. Therefore a transition from α-helix to β-sheet occurred in the protein structure during the fiber formation, although it is not known how and exactly when this change occurred. This is consistent with earlier findings with other silk proteins where β-sheet structure formation from disordered or other structures during fiber formation was thought to be responsible for the high tensile strength of silk fibers [32], [33]. Our findings are also consistent with earlier studies of proteins and fibers of natural wrapping silk, because protein content of the aciniform gland of *Nephila clavipes* was found to be mainly α-helical [23], while protein content in the natural wrapping silk fiber of *Nephila clavipes* was found to contain abundant β-sheet character and some helical character [34].

The introduction of recombinant spider wrapping silk protein AcSp1 may facilitate the development of useful biomaterials that differ from other artificial spider silks in certain properties. Among the different types of spider silks, dragline silk has the highest tensile strength, flagelliform silk has the highest elasticity, but wrapping silk has the highest toughness due to a combination of high tensile strength and high elasticity [25]. The W_4_ fibers are still not as strong and elastic as natural wrapping silk, which is most likely due to the fact that the W_4_ protein is much smaller than the native AcSp1 protein. Previously, artificial fibers formed from smaller dragline silk proteins also showed lower tensile strength and elasticity than artificial or natural fibers formed from native-sized dragline silk proteins [5], [29]. Fiber strength and elasticity may also be affected by fiber-forming conditions, as has been observed with other silk proteins [5], [15]. Therefore it should be possible to significantly increase the strength and elasticity of the W_4_ fibers by increasing the size of the recombinant protein and by devising a fiber-spinning method that includes post-drawing process. Conversely, variation and optimization of the fiber production process could lead to more native-like properties even with W_4_. As a whole, recombinant wrapping silk may provide new biomaterials of exceptional toughness, as already indicated by the high elasticity and a respectable tensile strength of the W_4_ fibers. The exceptionally small diameter of the W_4_ fibers can be another advantage for certain applications. For example, a very fine monofilament would be useful in microsurgery. Overall, our findings produced a new system for studying artificial fiber properties and formation, and may lead to the design and production of interesting biomaterials of desirable properties.

### Conclusion

We show that recombinant proteins derived from the spider wrapping silk protein AcSp1 can form silk-like fibers through shear-induced assembly in a physiological buffer. The fibers exhibited a relatively small (∼3.4 µm) diameter, high tensile strength and elasticity, which are reflective of the properties of natural wrapping silks. To assemble into silk fibers, the recombinant protein needed as few as two 200-aa AcSp1 repeats, contrary to 14 in the native spider silk. Furthermore, the C-terminal conserved non-repetitive domain was not required. Fiber formation appeared to involve an assembly of the recombinant proteins into microspheres, nanofibrils and silk-like fibers. Protein secondary structure determination indicated a transition from predominantly α-helical in solution to increasingly β-sheets in fibers. Our study unveils a new system for studying self-assembly mechanisms of spider silk proteins in forming silk fibers, providing a unique opportunity for comparing two different classes (aciniform silk vs. major ampullate silk) of spider silk proteins, and has outstanding promise for the engineering of biomaterials with desirable properties.
